# Magnitude of preterm birth and associated factors Among mothers who gave birth in Debre Berhan comprehensive specialized hospital

**DOI:** 10.3389/fgwh.2024.1375196

**Published:** 2024-05-28

**Authors:** Yosef Kibret, Abebe Minda Bunie, Sadat Mohammed, Tilahun Deresse Tamene, Tadesse Mamo Dejene

**Affiliations:** ^1^ Department of Pediatrics, Debre Berhan Comprehensive Specialized Hospital, Debre Berhan, Ethiopia; ^2^ Department of Public Health Debre Berhan University, Asrat Woldeyes Health Science Campus, Debre Berhan, Ethiopia; ^3^ School of Medicine, Debre Berhan University, Asrat Woldeyes Health Science Campus, Debre Berhan, Ethiopia

**Keywords:** magnitude, prevalence, preterm birth, Debre Berhan, hospital, Ethiopia

## Abstract

**Background:**

Premature deliveries are a major public health issue, with high health, economic, and productivity costs associated with lengthy hospitalizations in neonatal critical care units. The goal of this study was to determine the number of premature births in Ethiopia's Debre Berhan Comprehensive Specialized Hospital and the factors that influence them.

**Methods:**

The Debre Berhan Comprehensive Specialized Hospital conducted an institution-based cross-sectional study between February and April 2020. A total of 325 study participants were selected using systematic random sampling. Face-to-face interviews using a pre-tested semi-structured questionnaire were used to collect data. For data entry and analysis, Epi data version 3.1 and SPSS version 20 were used. At a *P*-value of 0.2, bivariate logistic regression analysis was used to categorize candidate variables to the next level, and variables in multivariate logistic regression models with a *p*-value of 0.05 were considered statistically significant.

**Result:**

Preterm births accounted for 16.1% of all births at Debre Berhan Comprehensive Specialized Hospital. Cesarean section [AOR = 2.412; 95% CI (1.154, 5.0370)], twin pregnancy [AOR = 3.524; 95% CI (1.114, 11.150)], and maternal anemia during pregnancy [AOR = 3.124; 95% CI (1.417, 6.887)] were statistically significant associations with the outcome variable in the final logistic regression model.

**Conclusion and recommendation:**

Preterm birth was found to be greater in the study area than in the Global Action Report for Sub-Saharan Africa and a few other countries. Efforts should be made to prevent maternal health issues that lead to caesarean section, and all pregnant mothers should be supplemented with iron and folic acid as soon as feasible. This study suggests that there is still a gap in the field in terms of health service intervention.

## 1 Introduction

Preterm delivery is defined by the World Health Organization (WHO) as delivering a baby before it achieves normal gestation or before term pregnancy, which implies before the 37th week of pregnancy or within 259 days after a woman's last normal menstrual period (LNMP) (1).

The purpose of this study is to assess the magnitude of preterm birth and its associated factors among mothers who gave birth in the Debre Berhan comprehensive specialized hospital.Littele is known about the magnitude of the problem in the study area. This specific hospital was chosen because the hospital serve as a referral center and has resources for preterm birth infrastructure and facilities in the north shoa zone of Amhara regional state of Ethiopia all other hospitals in the zone are primary hospitals and they refered a preterm birth baby to the Debre Berhan Comprehensive specialized hospital.

Preterm birth affects over 15 million babies worldwide, resulting in substantial economic expenses and lost productivity as a result of protracted stays in Neonatal Intensive Care Units after admission, as well as increasing long-term health needs due to lifelong disability (2).

Preterm births are caused by spontaneous labor in two-thirds of cases, and labor is terminated for fetal or maternal reasons in one-third of cases. Furthermore, according to World Health Organization data, real rates of preterm birth in Africa and Asia have increased more than expected (3, 4) Preterm birth was more common in underdeveloped nations, notably in Sub-Saharan Africa, and had more long-term repercussions for newborns, including developmental delays, serious health risks, and a significant socioeconomic burden, which included their families (5).

This early delivery is the leading cause of infant sickness and mortality in Ethiopia, according to the Ethiopian Demographic Health Survey (EDHS 2016); nonetheless, it is an avoidable health concerns (6).

Preterm birth, also known as prematurity, has resulted in the world's worst living conditions with significant economic and psychosocial consequences. For instance, in the United Kingdom, preterm infants accounted for 75% of Neonatal Intensive Care Unit (NICU) admissions, which can be a signal that a problem that can be considered a problem in developed countries as well (7).

In sub-Saharan Africa, more than 60% of newborns are born prematurely, resulting in huge productivity losses and poor health outcomes. Preventing the burden of prematurity among newborn babies remains a major challenge, particularly in developing countries, and preterm babies are more vulnerable to other illnesses and are the second leading cause of infant mortality; therefore, efforts to prevent prematurity should be increased across the routine healthcare system (8, 9).

Preterm birth is a leading cause of numerous medical difficulties, as well as unquantifiable emotional and psychosocial repercussions, all of which contribute to a lower quality of life for both the child and their family. Preterm birth prevention and treatment, on the other hand, did not reach the poorest and most vulnerable areas, where the burden was the greatest (10, 11).

Preterm birth is caused by a variety of factors, including maternal and sociodemographic characteristics, obstetric history, medical factors, and newborn biological characteristics, such as twin pregnancy and congenital anomalies. However, one-third of neonatal deaths are caused by preterm birth, indicating that further research is needed to reduce the burden of this issue (12).

Regarding the magnitude of preterm birth, according to countries’ profiles, 11% of the babies worldwide were born too early, which contributed to 15 million premature babies in number; for example, although the United States and Brazil rank among the top ten countries with the largest level of preterm births, 60% of preterm births still occur in Sub- Saharan Africa, and a cross-sectional study conducted in Iran, Brazil, Pakistan, Eastern Ethiopia and Kenia reported that the prevalence of preterm birth was 5.1%, 11.5%, 21.64%, 12.3%, 18.3%, respectively (13–18).

In Ethiopia, in a study conducted at Jimma University Specialized Hospital, the prevalence of preterm birth among neonates was found to be 25.9%, and at Shire Suhl General Hospital, as well as another study conducted at Axum and Adwa Towns Public Hospital, the prevalence of preterm birth was 16.9% and 13.3%, respectively preterm birth was found to be 16.15 percent in Addis Ababa Healthcare Facilities (19–22).

Regarding Factors associated with preterm birth, studies conducted in different parts of the world reported that educational level, place of residence, marital status, income level, family size, the interval between birth, medical check-ups, high blood pressure, preeclampsia, and type of pregnancy (multiple pregnancies) were variables that have a relationship with preterm birth. In addition, method of delivery like operation (cesarean section) or normal vaginal delivery, anemia, and age over 35 years have been associated with different premature births depending on the causes of premature birth and can be viewed as multifactorial or have many factors (16, 23–29).

Regarding Medical-related factors, cross-sectional research conducted in Axum and Adwa found that exposure to malaria during pregnancy, as well as the occurrence of chronic diseases such as Diabets Melitus (DM) and heart problems, was correlated with preterm birth, while another study conducted at the Debre Tabor Health Institute found that being anemic, HIV positive, and having a mid-upper arm circumference (MUAC) of less than 24 cm were statistically important predictors of preterm birth in the current pregnancy (14, 17, 19, 30).

On the other hand regarding Fetal related factors, according to a study conducted in Brazil in 2016, multiple gestations increased the risk of prematurity by more than 16 times compared to singleton pregnancies, and another study found that having a congenital defect increased the risk of prematurity by almost three times. Moreover, according to a study conducted in Kenya and other areas, male babies have a higher risk of preterm births than female babies, according to a study conducted in Kenya and other areas (17, 19, 29–32).

## Materials and methods

### Study area

The study was conducted in Debre Berhan Comprehensive Specialized Hospital, which was located in the Debre Berhan town North Shoa Zone Administration, Amhara National Regional State, Ethiopia. The town is the capital city of the North Shoa Zone, located 130 km and 695 km from Addis Ababa (the capital city of Ethiopia) and Bahir Dar (the Capital City of Amhara National Regional States), respectively.

Debre Berhan Comprehensive Specialized Hospital is one of Ethiopia's oldest public hospitals founded in 1929. It is currently the only Comprehensive Specialized Hospital in the North Shoa Zone administrative area, serving a catchment population of approximately 3 million people with a total staff of 652 people (426 health professionals, including 20 specialists and 226 administrative staff).

According to the 2018/2019 Hospital Annual Report, there were 170,800 outpatients, 8,640 inpatients, and 3,661 deliveries with a limited non-electronic record system. The Hospital had approximately eight wards with 154 inpatient beds. The obstetrics ward is one of the wards in the hospital, which has 13 postnatal beds, four 1st stage beds, and four coaches for 2nd stage laboring mothers with 35 health professionals, including four gynecologists.

### Study design

An institution-based cross-sectional study was conducted to assess the magnitude of preterm birth and its associated factors in Debre Berhan comprehensive specialized hospital. The data collection was completed from 02/01/2020 to 05/30/2020.

### Populations

The inferring population consisted of all mothers who gave birth at Debre Berhan Comprehensive Specialized Hospital during the study period, while the study population consisted of all mothers who gave birth at Debre Berhan Comprehensive Specialized Hospital throughout the study period. The study participants were also delivered to mothers interviewed in our sample.

### Inclusion and exclusion criteria

#### Inclusion criteria

All mothers who gave birth at the Debre Berhan Comprehensive Specialized Hospital during the study period.

#### Exclusion criteria

Mothers with an unknown last menstrual period (LNMP) and mothers who are absent for 1st-trimester ultrasound check-ups or have no ultrasound results were excluded from the study because it is difficult to determine the exact gestation of the baby and further will be difficult to separate the outcome as preterm or not.

### Sample size determination

To determine the sample size for this study, a single-population proportion formula was used. Considering the proportion of preterm birth from a previous similar study conducted in Jimma, Sothern, Ethiopia *p* = 25.9% (32) at a 95% confidence level and 5% margin of error.

$$\frac{n\;=\;{\left(Z\alpha /2\right)}^{2}p\;(1-p)}{d^{2}}$$

Where

• *n* = desired sample size
• Z = 95% Confidence limit i.e., 1.96
• *P* = 25.9% (proportion of the population which was taken from a previous study in Jimma)
• d is the margin of error or the desired degree of accuracy (0.05).
• 10% = is non respondent rate

By using single population proportion formula; the sample size was calculated as:

*n* = (1.96) (1.96) 0.259 (1-0.259)/(.05)^2^ = 294.9–295, then add 10% no response rate = 29.5–30, then by adding a non-response rate, the total sample size = 295 + 30 **= **325.

### Sampling technique and procedure

The number of mothers who gave birth regularly during the data collection period was used to calculate the duration of the data collection period and sampling value. The delivery registration book was checked at the time of data collection to determine the average birth rate per day, which was estimated to be 10 births per day.

The approximate total number of deliveries within three months of data collection was 900; therefore, the sample was selected using a systematic random sampling technique from the logbook with a sampling interval (K^th^) value (*k* = *N*/*n*).

Where ‘*N*’ was the total population by considering the study period (*N* = 900) and the minimum sample size which was (*n* = 325). Then, the value of *K* = *N*/*n* = 900/325 = 2.77≈3, every third delivery was taken to the sample by a systematic random sampling technique until the total minimum required sample was achieved.

The chosen mother was interviewed shortly after birth, according to the order of birth registers. The first mother to be included in the study was chosen at random, and every third mother was interviewed.

## Study variables

### Dependent variable or the outcome variable of this study is pre-term birth

#### Independent variables

##### Socio-demographic characteristics

Maternal age, residence, marital status, maternal religion, maternal educational status, maternal occupation, average household income, and family size.

##### All maternal obstetric variables

Included history of gravidity, previous abortion, previous stillbirth, previous preterm birth, history of ANC follow-up, number of ANC follow-ups, initiation of labor, premature rupture of membrane (PROM), mode of delivery, pregnancy-induced hypertension (PIH), and vaginal bleeding throughout the current pregnancy (APH).

##### Maternal medical history

Including diabetes mellitus (DM), heart disease, malaria during pregnancy, urinary tract infection (UTI), maternal recent hemoglobin level, maternal MUAC during pregnancy, maternal current stereo-status, and fetal factors such as multiple gestations, sex of babies, and congenital anomalies of the current baby are independent predictors of the outcome variable.

### Data collection and quality

Daily data were collected from sampled mothers in the postnatal ward through face-to-face interviews using a pre-tested semi-structured questionnaire.

#### Measurements

The non-stretchable MUAC tapes of the World Food Program were used to measure the left middle upper arm diameter, and an MUAC of less than 24 cm was chosen for this analysis. The record review aimed to gather information on variables related to mothers and neonatal health conditions.

Finally, LNMP and ultrasound results during the first trimester were used to assess gestational age because they yielded more accurate results. Preterm birth was defined as any neonate born at a gestational age of greater than 28 weeks but less than 37 weeks.

Following a review of the relevant literature, data collection tools were developed, written in English, translated into Amharic, and then translated back into English to ensure consistency. Two diploma midwives and one degree midwife personnel served as data collectors and supervisors, respectively.

For two days, all data collectors and supervisors received adequate training on the importance of the analysis, purpose of the study, sampling methodology, and collection of data to produce a successful report. The questionnaire was pre-tested on 5% of the sample size in Deneba Primary Hospital among eligible mothers to determine the tool's reliability and to check the questioner's kipping pattern and content.

The results were discussed among the data collectors and supervisors so that the tool could be updated before the actual data collection began, and the final interview could then be performed at a suitable time using a modified questionnaire. The principal investigator and supervisor checked the completeness of the data regularly during the data collection period and corrective steps were taken when a problem was discovered.

## Data analysis

Because the outcome variable in this study is binary (preterm birth, yes or no), logistic regression was the model of choice to determine the magnitude of preterm birth and its associated factors. SPSS version 20 software were used for the analysis of the data. The forward type of logistic regression was used to test the independent variables and demonstrate their association with the outcome variable at the *p* < 0.05 level of significance with 95% confidence interval.

## Result

### Socio-demographic descriptions of the respondents

The Study targeted 325 mothers. However, 316 mothers were enrolled with a response rate of 97.2%. The mean age of the mothers who participated was 28.86 years with a deviation of 6 years (SD ± 6.3 years).

The majority 281 (88.9%), 265 (83.9%), and 212 (67.1%) of the study participants were married, Ethiopian orthodox Christen followers, and live-in urban residents, respectively. More than half 172 (54.4%) of mothers attended secondary and above level of education and 166 (52.5%) were unemployed mothers. Two hundred twenty (69.6%) of the respondent's household monthly income was ≤5,000 Ethiopian Birr, and about 63.9% of the respondents had ≥four family members in the household (Table 1).

**Table 1:** Socio-demographic characteristics of the respondents in Debre Berhan comprehensive specialized hospital, North Shoa, Amhara, Ethiopia, 2020 (*n* = 316).

Variables	Frequency	Percent %
Age of mothers		
≥35	67	21.2
<35	249	78.8
Maternal residency		
Rural	104	32.9
Urban	212	67.1
Maternal marital status		
Single	35	11.1
Married	281	88.9
Maternal religion		
Orthodox	265	83.9
Muslim and others	51	16.1
Maternal education		
Primary or less	144	45.6
Secondary and above	172	54.4
Maternal occupation		
Unemployed	166	52.5
Employed	150	47.5
Monthly household income		
≤5,000	220	69.6
>5,000	96	30.4
Family size		
≥4	202	63.9
<4	114	36.1

### Maternal obstetric history

Out of 316 mothers, 205 (64.9%) of them were multigravida mothers regarding the antenatal care visits, 294 (93%) of the respondents had ANC follow-up, and more than half 196 (62.0%) had four or more ANC visits, which are recommended antenatal care visits. The majority of the study participants 205 (64.9%) have a history of previous pregnancy, 67 (21.2%) had a history of previous abortion in addition to this 27 (8.5%) had a history of previous preterm birth. Regarding the types of labor and mode of the delivery majority of them had spontaneous onset of labor 274 (86.7%) and spontaneous vaginal delivery 200 (63.3%) but, the rest of mothers had induced onset of labor 42 (13.3%) and delivered via cesarean section 116 (36.3%). The majority of delivered mothers never had Anti Partum hemorrhage, which is bleeding during the pregnancy period, Pregnancy Induced hypertension, and premature rupture of membranes medically known as PROM during their current pregnancy 312 (98.7%), 284 (89.9%), and 232 (73.4%), respectively) (Table 2).

**Table 2:** Maternal obstetrics-related factors of the respondents in Debre Berhan comprehensive specialized hospital, North Shoa, Amhara, Ethiopia, 2020 (*n* = 316).

Variables	Frequency	Percent (%)
History of pregnancy		
Yes	205	64.9
No	111	35.1
History of abortion		
Yes	67	21.2
No	249	78.8
History of still birth		
Yes	44	13.9
No	272	86.1
History of preterm birth		
Yes	27	8.5
No	289	91.5
History of ANC visits		
Yes	294	93.0
No	22	7.0
Number of ANC visits		
<4 ANC	120	38.0
≥4 ANC	196	62.0
Types of labor started		
Induced	42	13.3
Spontaneous	274	86.7
Pre-rupture of membrane		
Yes	84	26.6
No	232	73.4
Mode of delivery		
C/S	116	36.7
SVD	200	63.3
Pregnancy induced hypertension		
Yes	32	10.1
No	284	89.9
Anti-partum hemorrhage		
Yes	4	1.3
No	312	98.7

### Medically related factors of the mothers

Out of the 316 delivered mothers, 60 (19%) had urinary tract infections. Concerning the HIV status of the mother, all of them were tested for HIV infection and 23 (7.3%) were positive for the infection; only seven mothers had malaria during the current pregnancy regardless of malnutrition; all respondents measured their left Mid-Upper Arm circumference and one, 105 (33.2%) mothers were malnourished.All participants had their haemoglobin level checked and 49 (15.5%) of mothers had anaemia (Table 3).

**Table 3:** Medical-related factors of the study participants in Debre Berhan comprehensive specialized hospital, North Shoa, Amhara, Ethiopia, 2020 (*n* = 316).

Variables	Frequency	Percent %
Diabetic mellitus		
Yes	1	0.3
No	315	99.7
History of heart disease		
Yes	5	1.6
No	311	98.4
Malaria during pregnancy		
Yes	7	2.2
No	309	97.8
Urinary tract infection		
Yes	60	19.0
No	256	81.0
Maternal Hgb Level		
<11	49	15.5
≥11	267	84.5
Maternal HIV status		
Positive	23	7.3
Negative	293	92.7
Maternal left MUAC		
<24	105	33.2
≥24	211	66.8

### Newborn characteristics

**Figure 1:**
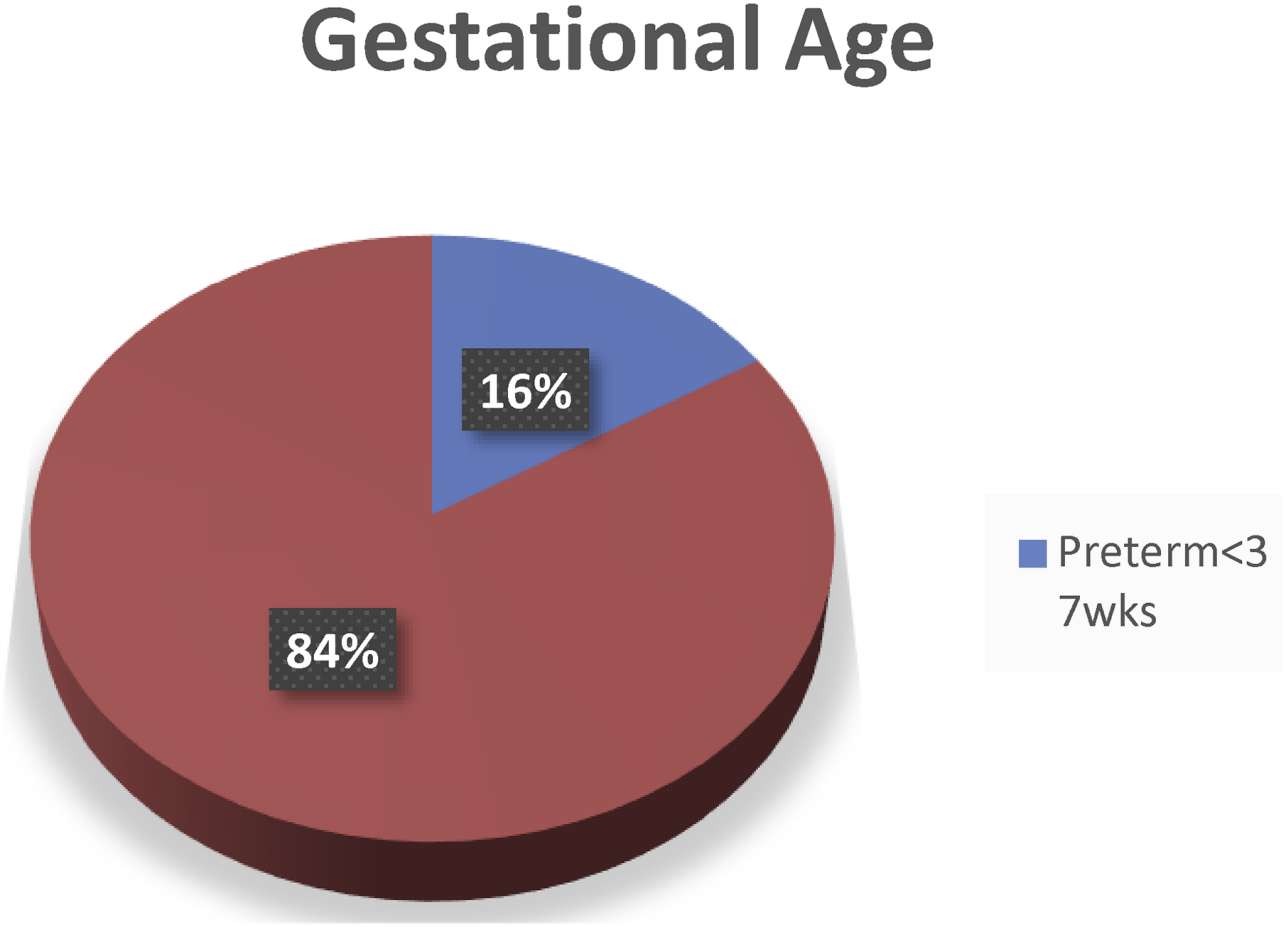
Gestational age of the newborn in Debre Berhan comprehensive specialized hospital, North Shoa, Amhara, Ethiopia, 2020.

The majority of the newborns 297 (94.3%) were single, the remaining 18 (5.7%) were twins, and 32(10.1%) of the newborns had some sort of congenital anomaly. Regarding the sex of the newborn, more than half 176 (55.7%) were male babies. The majority of the newborns babies 265 (83.9%) were not pre-term (Figure 1).

## Factors associated with preterm birth

Predictor variables were tested using a binary logistic regression model, and variables with a *p*-value <0.2 were transferred to run in multivariate logistic regression. When controlling for confounding variables in multivariate analysis, we found that mode of delivery (C/S), type of pregnancy (twin pregnancy), and maternal anemia had a statistically significant association with pre-term birth (*p* < 0.05). The effect of correlations among independent predictors (multicollinearity) was assessed, and Hosmer–Lemeshow's goodness of fit was checked for fitting these models (0.189).

Mothers who delivered by caesarean section were 2.4 times more likely to have a pre-term birth than those who delivered by Spontaneous Vaginal Delivery (AOR = 2.416; 95%CI: 1.154–5.037). Mothers who had twin pregnancies were more than 3.5 times more likely to give pre-term birth as compared to singleton pregnancies (AOR = 3.524, 95%CI: 1.114–11.150). Anaemic pregnant mothers were more than 3.5 times more likely to have preterm birth as compared to mothers who were not anaemic (AOR = 3.124; 95%CI: 1.417–6.887) (Table 4).

**Table 4:** Bivariate and multivariate analyses among selected factors associated with preterm birth mothers who delivered in DBCSH, North Shoa, Amhara, Ethiopia, 2020 (*n* = 316).

Variables	Pre term birth		COR (95% CI)	AOR (95%CI)
	Yes	No		
Residency				
Rural	22 (43.1%)	82 (30.9%)	1.693[.918–3.123]	0.877[.385–2.002]
Urban	29 (56.9%)	183 (69.1%)	1.0	1.0
History of abortion				
Yes	16 (31.4%)	51 (19.2%)	1.918[.986–3.732]	1.394[.626–3.105]
No	35 (68.6%)	214 (80.8%)	1.0	1.0
History still birth				
Yes	11 (21.6%)	33 (12.5%)	1.933[.904–4.136]	0.841[.297–2.381]
No	40 (78.4%)	232 (87.5%)	1.0	1.0
History of preterm				
Yes	7 (13.7%)	20 (7.5%)	1.949[.778–4.883]	0.849[.266–2.705]
No	44 (86.3%)	245 (92.5%)	1.0	1.0
ANC follow up				
Yes	45 (88.2%)	249 (94.0%)	0.482[.179–1.298]	0.47[.166–1.809]
No	6 (11.8%)	16 (6.0%)	1.0	1.0
No ANC follow up				
<4	25 (49.0%)	95 (35.8%)	1.721[.941–3.147]	1.911[.854–4.273]
≥4	26 (51.0%)	170 (64.2%)	1.0	1.0
Mode of delivery				
C/S	25 (49.0%)	91 (34.3%)	1.839[1.004–3.366]	2.412[1.154–5.037]*
SVD	26 (51.0%)	174 (65.7%)	1.0	1.0
History of PIH				
Yes	11 (21.6%)	21 (7.9%)	3.195[1.432–7.129]	2.500[.986–6.337]
No	40 (78.4%)	244 (92.1%)	1.0	1.0
Heart disease				
Yes	2 (3.9%)	3 (1.1%)	3.565[.580–21.891]	3.412[.787–37.229]
No	49 (96.1%)	262 (98.9%)	1.0	1.0
Pregnancy type				
Multiple	7 (13.7%)	11 (4.2%)	3.674[1.351–9.988]	3.524[1.114–11.150]*
Singleton	44 (86.3%)	254 (95.8%)	1.0	1.0
Hemoglobin				
<11	18 (35.3%)	41 (15.5%)	3.214[1.604–6.439]	3.124[1.417–6.887]*
≥11	33 (64.7%)	224 (84.5%)	1.0	1.0
MUAC				
<24	21 (41.2%)	84 (31.7%)	1.508[.816–2.789]	1.744[.859–3.545]
≥24	30(58.8%)	181(68.3%)	1.0	1.0

1.0, reference category, *statistically significant at *P* < 0.05.

## Discussion

The study was to examine the association between pre-term births and maternal and newborn characteristics in the Debre Berhan Comprehensive Specialized Hospital. The gestational age of the newborn neonate was utilized as a cut-off point to determine the outcome variable, which was less than 37 completed weeks of gestation, but after viability (>28 weeks).

Pre-term birth was determined to be 16.1 percent in this study, with a 95 percent confidence interval of (11.4–19.9), which is higher than the 12.3 percent identified in the Global Action Report for Sub-Saharan African countries (33) and study in Brazil (2016). Dodola, Axum, and Gondar town health institutions showed that (11.5%), ((13%), (13.3%), and (4.4%), respectively (17, 19, 25, 27).

This difference is most likely due to differences in study settings and study areas, or it could be because our study area is at a higher risk than others due to various health-related gaps, such as service quality, or it could be due to differences in health-related medical illnesses and pregnancy-related histories of participants from other areas. On the other hand, studies may differ in settings, such as one study excluding multiple gestations and another excluding those with a history of abortion; however, surgical evacuation of the uterus mechanically stretches the cervix, predisposing such mothers to preterm birth in subsequent pregnancies; thus, study settings may be the reason for the discrepancies observed. However, the results of this study did not differ from those of other studies conducted in Ethiopia, such as those conducted in Addis Ababa public hospitals and the Sehul General Hospital, which reported preterm birth rates of 16.15 percent and 16.9 percent, respectively (26, 34).This similarity in magnitude could be because the two studies mentioned used similar methods to selected study subjects.

The current study's findings were lower than those of a cross-sectional study conducted in Kenya National Hospital and Jemma University Specialized Hospital, which found that preterm delivery was prevalent in 20.2 percent and 25.9 percent, respectively (22, 29) This difference could be attributed to a difference in study time, indicating that the Federal Ministry of Health (FMOH) is now providing better maternal health care than in the past. Another reason for this variation could be differences in the study area. A study conducted in Kenya and Jimma found that the high prevalence of alcohol and substance intake during pregnancy is another contributing factor to increased preterm birth magnitudes in their study area, but this was not the case in our study.

This study found a statistically significant association between cesarean delivery and premature birth. Compared to mothers who delivered via spontaneous vaginal delivery, mothers who delivered via cesarean section had a 2.4 higher risk of premature birth. This conclusion was supported by research conducted in Brazil and Kenya (17, 22). Even if surgical delivery did not have a direct causative relationship with pre-term birth, these groups of mothers were determined to be in the category of premature birth in our study as a result of the medical indication of operation or caesarian delivery conducted before normal labour. Certain maternal or fetal disorders require immediate attention as life-saving interventions. They are also suspected to be a factor in the increased caesarean delivery rates before labor.

In this study, twin pregnancies were associated with pre-term births. Mothers who had twin pregnancies had 3.5 increased odds of pre-term delivery than those mothers who delivered a single baby. This study was consistent with the finding of the studies in Malaysia, Cameron, Kenya, and Axum, respectively (19, 20, 22, 34). Multiple pregnancies are a known predisposing factor for premature birth due to uterine over distension and stretching of the myometrium, encouraging the induction of oxytocin receptors, leading to early onset of uterine contractions and spontaneous preterm birth. Obstetric complications, such as pregnancy-induced hypertension and APH, are common problems in twin pregnancies, which require surgical delivery and increase the risk of preterm birth (35).

Anemic mothers were more than three times more likely to have preterm births than non-anemic mothers. This figure is in line with the findings of Shire and Debre Tabor (21, 32). This might be due to the decreased blood flow to the placenta, which results in placental insufficiency. Physiologically, haemoglobin is transferred from the mother to the fetus through the interface of the placenta; however, when the blood supply decreases, the placenta detaches from the uterine wall, leading to premature labor and delivery. In a normal functioning body, haemoglobin is used as a mine for oxygen transportation to the whole body; therefore, a low Hgb level can cause hypoxia (lack of oxygen or shortage) that increases maternal and fetal stress, which initiates the production of corticotrophin-releasing hormone and leads to the initiation of pre-term labor. Normally, at the time of pregnancy, haemoglobin levels rapidly decrease to balance the blood volume and fit the iron needs of the fetus (36).

### Limitation of the study

Because the study was conducted at a single institution, the North Shoa Zone referral hospital, the study design was cross-sectional in nature, making it unable to demonstrate the casual relationships between the variables. As a result, additional research at the national and regional levels is needed to strengthen the findings.

## Conclusion and recommendation

This study showed the magnitude of preterm be higher than that of the Global Action Report for Sub-Saharan Africa and some other areas of study. Evidence from this study found that the mode of delivery, multiple gestation, and maternal anemia during recent pregnancy were significantly associated with preterm birth. In summary, the findings of this study showed that preterm delivery is a major public health issue, particularly in the study area.

The Hospital should address iron and folate supplements for all mothers as early as possible before and during pregnancy and strengthen early screening of anemia and high-risk pregnancy, such as multiple gestations. Avian blood bank access is used to manage complications of anemia as early as possible, and it would be better if health professionals watch out and mitigate mothers with problems that might lead to cesarean section. In addition, it would be better if they designed clinical protocols that needed appropriate indications for cesarean section rather than simply trying to remember from different sources of medical science alone.

## Data Availability

The raw data supporting the conclusions of this article will be made available by the authors, without undue reservation.
